# Cell–cell contacts prevent *t*-BuOOH-triggered ferroptosis and cellular damage in vitro by regulation of intracellular calcium

**DOI:** 10.1007/s00204-024-03792-5

**Published:** 2024-05-30

**Authors:** Dagmar Faust, Christine Wenz, Stefanie Holm, Gregory Harms, Wolfgang Greffrath, Cornelia Dietrich

**Affiliations:** 1 Institute of Toxicology, University Medical Center of the Johannes Gutenberg University, Obere Zahlbacher Straße 67, 55131 Mainz, Germany; 2 Department of General and Visceral Surgery, Albklinik Münsingen of the District Hospital Association Reutlingen, Lautertalstraße 47, 72525 Münsingen, Germany; 3 Cell Biology Unit, University Medical Center of the Johannes Gutenberg University, Langenbeckstraße 1, 55131 Mainz, Germany; 4 Department of Neurophysiology, Mannheim Center for Translational Neuroscience (MCTN), Heidelberg University, Ludolf-Krehl-Straße 13-17, 68167 Mannheim, Germany

**Keywords:** *t*-BuOOH, Calcium, Ferroptosis, Toxicity, Cell–cell contacts

## Abstract

*Tert*-butyl hydroperoxide (*t*-BuOOH) is an organic hydroperoxide widely used as a model compound to induce oxidative stress. It leads to a plethora of cellular damage, including lipid peroxidation, DNA double-strand breaks (DNA DSBs), and breakdown of the mitochondrial membrane potential (MMP). We could show in several cell lines that *t*-BuOOH induces ferroptosis, triggered by iron-dependent lipid peroxidation. We have further revealed that not only *t*-BuOOH-mediated ferroptosis, but also DNA DSBs and loss of MMP are prevented by cell–cell contacts. The underlying mechanisms are not known. Here, we show in murine fibroblasts and a human colon carcinoma cell line that *t*-BuOOH (50 or 100 µM, resp.) causes an increase in intracellular Ca^2+^, and that this increase is key to lipid peroxidation and ferroptosis, DNA DSB formation and dissipation of the MMP. We further demonstrate that cell–cell contacts prevent *t*-BuOOH-mediated raise in intracellular Ca^2+^. Hence, we provide novel insights into the mechanism of *t*-BuOOH-triggered cellular damage including ferroptosis and propose a model in which cell–cell contacts control intracellular Ca^2+^ levels to prevent lipid peroxidation, DNA DSB-formation and loss of MMP. Since Ca^2+^ is a central player of toxicity in response to oxidative stress and is involved in various cell death pathways, our observations suggest a broad protective function of cell–cell contacts against a variety of exogenous toxicants.

**Supplementary Information:**

The online version contains supplementary material available at 10.1007/s00204-024-03792-5.

## Introduction

Cell–cell contacts control embryonic development and maintain tissue homeostasis in the adult organism. They are crucial regulators of cellular proliferation, differentiation, organ size control and migration in vitro and in vivo (Dietrich et al. 1997; Eagle and Levine 1967; Faust et al. 2005; Heit et al. 2001; Kang and Massague 2004; Weiss et al. 2008; Zhao et al. 2007). It has also been shown that cell–cell contacts control sensitivity to chemotherapeutics and cell death (Bar et al. 2004; Gujral and Kirschner 2017). The different types of cell–cell contacts include adherens junctions, desmosomes, and tight junctions. In epithelial cells, adherens junctions are built up by E-cadherin dimers, whereas N-cadherin is a major cell adhesion molecule in non-epithelial cells, such as fibroblasts and neurons (Derycke and Bracke 2004; Yap et al. 1997). They affect cellular signaling by inhibiting or stimulating several master pathways, such as the Wnt pathway, receptor tyrosine kinase signaling, and the Hippo pathway, respectively [for review, see (Dongre and Weinberg 2019; Huang et al. 2019)].

Recent evidence suggests that cell–cell contacts also regulate ferroptosis [for review, see (Dietrich and Hofmann 2021; Vucetic et al. 2020)]. Ferroptosis is a regulated cell death pathway characterized by two hallmarks: overwhelming phospholipid peroxidation and dependence on iron (for review see Chen et al. 2020; Doll and Conrad 2017; Hassannia et al. 2019; Jiang et al. 2021). Ferroptosis lacks typical features of previously recognized cell death forms, such as apoptosis, autophagy, or necroptosis, which are caspase activation, autophagic vesicle formation, or receptor-interacting serine/threonine–protein kinase 3 (RIPK3)-activity, respectively (Dixon et al. 2012). Elucidating the regulation of ferroptosis is attractive for two therapeutic reasons: prevention of organ damage mediated by ferroptosis, e.g., in ischemia reperfusion injury, drug-induced liver failure, Parkinson’s or Alzheimer’s disease and, vice versa, induction of ferroptosis to improve cancer therapy (Conrad et al. 2021; Lei et al. 2022; Stockwell 2022; Tan et al. 2022; Zhang et al. 2022). Ferroptosis can be induced by suppression of defense systems or increased formation of phospholipid hydroperoxides [for review, see (Hassannia et al. 2019)]. A classical trigger is the small compound erastin, which inhibits the cystine/glutamate exchanger system x_c_^−^ leading to a decrease in cystine intake, thereby cysteine availability and finally a decrease in GSH levels, a critical cofactor of glutathione peroxidase 4 (GPX4) (Dixon et al. 2012). GPX4 reduces lipid hydroperoxides to lipid alcohols and, therefore, is a critical enzyme in phospholipid detoxification. However, ferroptosis can also be evoked by several other mechanisms (Bersuker et al. 2019; Doll et al. 2019; Friedmann Angeli et al. 2014; Hassannia et al. 2018; Torii et al. 2016; Yang and Stockwell 2008). We have recently found that the organic hydroperoxide *t*-BuOOH induces ferroptosis in a panel of murine and human cell lines (Wenz et al. 2018). *t*-BuOOH is a widely used model compound to induce oxidative stress in vitro and in vivo, and is considered to imitate the effects of physiological short-chain lipid hydroperoxides (Ayala et al. 2014). However, the precise molecular mechanisms of *t*-BuOOH-mediated toxicity are not fully understood. Beyond ferroptosis, *t*-BuOOH leads to formation of DNA double-strand breaks (DSBs) and loss of the mitochondrial membrane potential (MMP), but ferroptosis is executed independently of these damages (Wenz et al. 2018). We could also show that cell–cell contacts protect against *t*-BuOOH-induced ferroptosis in a panel of murine and human cell lines, and we hypothesized that the cell adhesion molecules E- or N-Cadherin, respectively, might regulate ferroptosis (Dietrich and Hofmann 2021; Wenz et al. 2019). It was then made evident by Wu and co-workers that, indeed, E- or N-Cadherin mediate protection by activating the Hippo pathway (Wu et al. 2019). The authors show that E/N-Cadherin activate Merlin which promotes phosphorylation of large tumor suppressor kinase 1 (LATS) leading to phosphorylation of the transcriptional coactivator YAP and its nuclear exclusion. As a result, expression of acyl-CoA synthetase long-chain family member 4 (ACSL4) and the transferrin receptor 1 (TfR1), both important regulators of ferroptosis, is decreased. However, we had also demonstrated, that cell–cell contacts do not only prevent ferroptosis, but also inhibit *t*-BuOOH-induced DNA DSBs as well as dissipation of the MMP. Moreover, cell–cell contacts protect against non-ferroptotic cell death induced by hydrogen peroxide and against apoptosis mediated by methyl methanesulfonate or UV-C (Wenz et al. 2019). These observations argue for a broader protective function of cell–cell contacts and additional intracellular mechanisms beyond regulation of ACSL4 and TfR1. Here, we present a novel protective mechanism involving Ca^2+^ as a central regulator. This pathway does not involve downregulation of ACSL4 or TfR1. We demonstrate that *t*-BuOOH leads to an increase in intracellular Ca^2+^ levels ([Ca^2+^]_i_) in subconfluent cultures, and that Ca^2+^ is required not only for lipid peroxidation and ferroptosis, but also for induction of DNA DSBs and dissipation of the MMP. In confluent cells, *t*-BuOOH-mediated accumulation of intracellular Ca^2+^ is strongly blocked, hence preventing lipid peroxidation and ferroptosis, DNA DSB formation and loss of MMP. We provide a model in which cell–cell contacts regulate intracellular Ca^2+^ levels thereby protecting from ferroptosis and ROS-induced cellular damage.

## Materials and methods

### Test chemicals and cell culture

Test chemicals. *t*-BuOOH, CoCl_2_, polyethylene glycol 8000 (Sigma-Aldrich, St. Louis, MO, USA), BAPTA-AM, liproxstatin-1 (Cayman, Ann Arbor, MI, USA), ionomycin (Calbiochem, Darmstadt, Germany), EGTA (ethylene glycol-bis(ß-aminoethyl ether)-N,N,N′,N′-tetraacetic acid, Carl Roth, Karlsruhe, Germany).

Cell culture. The non-transformed murine fibroblast cell line NIH3T3 (Cell Line Service CLS, Eppelheim, Germany) was routinely cultured in Dulbecco’s Modified Eagle’s Medium (DMEM, Gibco Life Technologies, Carlsbad, CA, USA), supplemented with 4 mM glutamine, penicillin, streptomycin (each 100 U/mL, Sigma-Aldrich), and 10% fetal bovine serum (FBS, Sigma-Aldrich). The human colon carcinoma cell line Caco-2 (Cell Lines Service CLS) was routinely cultured in Modified Eagle’s Medium (MEM, Sigma-Aldrich) additionally supplemented with non-essential amino acids (Sigma-Aldrich). Cells were kept at 37 °C in a humidified atmosphere containing 5% CO_2_. Cells were either sparsely seeded (NIH3T3: 1.5 × 10^4^ cells/cm^2^, Caco-2: 1 × 10^4^ cells/cm^2^) or seeded to confluence (NIH3T3: 1.2 × 10^5^ cells/cm^2^, Caco-2: 8 × 10^4^ cells/cm^2^) (Wenz et al. 2019), cultured for 24 h and then treated as described in the figure legends.

### Determination of cell death

Cells were washed, trypsinized, and pelletized together with the supernatant by centrifugation. After repeated washing, the cell pellet was resuspended in 200 µL of phosphate-buffered saline (PBS) containing propidium iodide (PI) (1.25 µg/mL, AppliChem, Darmstadt, Germany). 10.000 cells per sample were scored and PI-positive cells were determined by BD CellQuest Pro (FACS Calibur) (BD Becton Dickinson, Heidelberg, Germany).

### Western blot analysis

Cells were lysed in hot Laemmli sample buffer (Laemmli 1970). Protein concentration was determined according to (Smith et al. 1985). Equal amounts of protein (20 µg per lane) were separated by SDS–PAGE (10%) and electroblotted onto Immobilon membranes (Merck Millipore, Darmstadt, Germany). The blots were blocked with 5% low-fat milk powder in Tris-buffered saline (TBS, 50 mM Tris–HCl, pH 7.5, 150 mM NaCl) containing 0.1% Tween 20 for 1 h and then incubated with anti-Transferrin Receptor 1- (1 µg/mL, ab84036) or anti-ACSL4-antibody (1:5000, ab155282, both Abcam, Cambridge, UK) at 4 °C overnight, followed by incubation with horseradish peroxidase-conjugated secondary antibody and ECL-detection (both Cell Signaling, Beverly, MA, USA) according to the manufacturer’s instructions. To control for equal loading, the blots were stripped and reprobed with anti-ß-actin-antibody (1:1000, sc-47778, Santa Cruz, Dallas, TX, USA) followed by ECL-detection.

### Measurement of intracellular Ca^2+^

Intracellular Ca^2+^ was measured by the cell-permeable fluorescent calcium indicator Fluo-4 acetoxymethyl ester (Fluo*-*4-AM, ThermoFisher Scientific, Waltham, MA, USA). Cells were treated as described in the figure legends. Cells were washed with PBS, covered with Phenol red-free DMEM (Gibco Life Technologies) containing 2 µM Fluo-4-AM and 0.02% Pluronic F-127 (ThermoFisher Scientific) at 37 °C for 30 min. Cells were then washed with PBS, trypsinized, and pelletized by centrifugation. Cells were resuspended in PBS and flow cytometric analysis was performed by a FACS CantoII (BD Becton Dickinson).

Alternatively, changes of free intracellular Ca^2+^ ([Ca^2+^]_i_) were measured over time using life cell imaging technique. After seeding on poly-L-lysine-covered (10 µg/mL, Sigma-Aldrich) round microscope cover slips (diameter 15 mm; Carl Roth) cells were loaded with 2 µM of the fluorescent ratiometric calcium indicator Fura 2-AM (prepared as stock solution: 1 mM in DMSO, Biotrend, Cologne; Germany) in Tyrode’s solution (1.8 mM CaCl_2_, 5 mM glucose, 10 mM HEPES, 5.4 mM KCl, 0.5 mM MgCl_2_, 137.6 mM NaCl, pH 7.3) for ~ 30 min at room temperature in the dark (20–25 °C). After washing with Tyrode, the coverslip was mounted in an open bath chamber (Series 40 Quick Change Imaging Chamber; Warner Instruments, Hamden, USA). For imaging, an inverted fluorescence microscope (Olympus IX81; Olympus, Tokyo, Japan) and an image acquisition and analysis system (xcellenceRT; Olympus) were used. All experiments were carried out at room temperature in the dark. Cells were alternatively excited with light of 340 and 380 nm wavelength (for 80 ms, each) once a minute. The respective fluorescence intensities at 510 nm were recorded with an ORCA-R2 CCD camera (Hamamatsu Photonics, Hamamatsu, Japan). The ratio between 510 nm fluorescence emissions for 340 nm excitation and 380 nm excitation was used as measure for the relative change in [Ca^2+^]_i_ (Grynkiewicz et al. 1985). After a short baseline period of 3 min, slow application of 2 mL of Tyrode was used to determine putative mechanical artifacts induced by superfusion; 3 min thereafter either 2 mL of 50 µM *t*-BuOOH or of the respective vehicle solution were added to the chamber and cells were observed for up to 6 h thereafter. Shortly before the experiments ended, 10 µM ionomycin (1 mM stock solution in DMSO) was applied as a positive control for an increase in [Ca^2+^]_i_.

### Detection of lipid ROS

Lipid peroxidation was measured by the lipid ROS probe Bodipy 581/591 C11 (Gibco Life Technologies). Cells were treated as described in the figure legends, washed with PBS, and stained with 2 µM Bodipy 581/591 C11 in Phenol red-free DMEM at 37 °C for 30 min. Cells were then washed with PBS, trypsinized, and pelletized by centrifugation. Cells were resuspended in PBS and flow cytometric analysis was performed by a FACS CantoII.

### Detection of DNA double-strand breaks

DNA double-strand breaks (DNA DSBs) were detected by the neutral Comet assay. About 10^4^ cells were mixed with 120 µL of low melting agarose (0.5%) (ThermoFisher Scientific) and transferred onto slides procoated with agarose (ThermoFisher Scientific). Lysis (2.5 M NaCl, 100 mM EDTA, 10 mM Tris, 1% Triton X-100, pH 7.5) was performed at 4 °C for 60 min. Cells were placed in an electrophoresis chamber and mounted in a neutral electrophoresis buffer (90 mM Tris, 2 mM EDTA, 90 mM boric acid, pH 7.4). After electrophoresis (25 V, 35 min), slides were washed with purified water, air-dried for 2 h with 100% ethanol (Carl Roth) and stained with 50 µg/mL PI. Comets were analyzed by fluorescence microscopy using an Olympus BX50 microscope equipped with a ColorView camera (Olympus). At least 50 cells/slide were scored using the Comet IV software (Perspective Instruments Ltd., Bury St Edmund, UK).

Alternatively, DNA DSBs were visualized by γ-H2AX-immunofluorescence. Cells were washed with PBS and fixed with ice-cold methanol/acetone (70%/30%) at − 20 °C for 8–9 min. Cells were then washed three times with PBS (3 × 5 min) followed by blocking with 10% normal goat serum (Merck)/0.25% Triton X-100 at room temperature for 1 h. Cells were then incubated with anti-γ-H2AX-antibody (1:1000, #9718, Cell Signaling) in PBS/0.25% Triton X-100 at 4 °C overnight. After washing cells with PBS (3 × 5 min), they were incubated with Alexa-Fluor 488-conjugated antibody (1:400, ThermoFisher Scientific) at RT for 1 h. After three washing steps with PBS, nuclei were counterstained with To-Pro-3 (1:100 in PBS, ThermoFisher Scientific). After washing, antifade medium (Vectashield, Vector Laboratories, CA, USA) was dropped onto clean slides and the cover slips were transferred onto the slides and fixed with nail polish. Representative confocal images were captured by a laser scanning microscope (LSM710, Carl Zeiss, Oberkochen, Germany).

### Loss of mitochondrial membrane potential (ψ∆m)

Loss of ψ∆m was detected by staining with DiOC6 (3,3′-dihexyloxacarbocyanine iodide) (ThermoFisher Scientific). Cells were treated as described in the figure legends. Cells were washed and stained with 20 nM DiOC6 in phenol red-free DMEM at 37 °C for 20 min. After trypsinization, cells were pelletized by centrifugation, and resuspended in PBS, followed by dropwise addition of propidium iodide to exclude necrotic cells from analysis. Analysis was performed by flow cytometry (FACS CantoII).

### Statistical analysis

Comparisons were made by one- or two-way analysis of variance (ANOVA) followed by Tuckey’s multiple comparison test. A *p* value of ≤ 0.05 was considered to be significant.

## Results

### The ferroptosis regulators TfR1 and ACSL4 are not downregulated at the time of t-BuOOH treatment

**Fig. 1:**
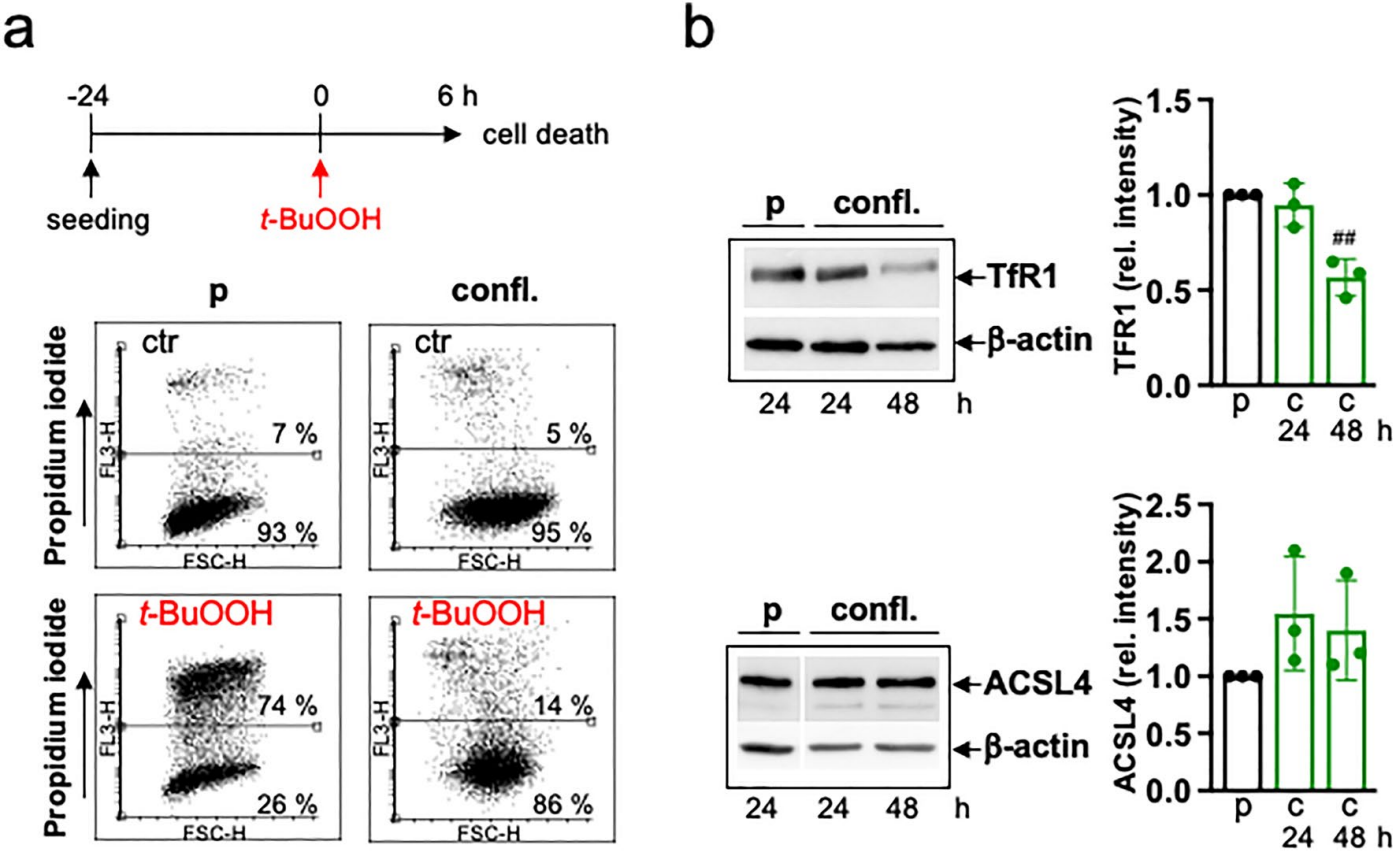
Cell–cell-contacts mediate protection against *t*-BuOOH-triggered ferroptosis, but TfR1 and ACSL4 are not downregulated at this time point. **a** NIH3T3 cells were either sparsely seeded (proliferating = p) or seeded to confluence (confluent = confl.) and cultured for 24 h. *t*-BuOOH (50 µM) was added for 6 h and cell death analyzed by PI staining and flow cytometry. Data are shown as a representative flow cytometry dot plot. **b** NIH3T3 cells were either sparsely seeded (proliferating = p) and cultured for 24 h or seeded to confluence (confluent = confl.) and cultured for 24 and 48 h. Total cell extracts were prepared and Western blotting performed using an anti-TfR1- or anti-ACSL4-antibody. Blots were stripped and reprobed with anti-β-actin-antibody to control equal loading. Left: representative blots. Right: quantification of three independent blots each, results presented as means ± SD (including individual data points) relative to control, ^##^*p* ≤ 0.01, significant inhibition

We have recently shown that confluent NIH3T3 cells and various other non-tumorigenic cell lines are protected against* t*-BuOOH-triggered ferroptosis (Fig. 1a) (Wenz et al. 2019) and cellular damage, i.e. loss of MMP and DNA DSBs (see below and (Wenz et al. 2019)). In our cell culture model, cells are seeded to confluence below their saturation density and treated with *t*-BuOOH 24 h after seeding. It is important to note that cell–cell contacts are just established, but the cells are not quiescent at the time point of treatment (Wenz et al. 2019). This was demonstrated by cell counting, cell cycle analysis and Western blotting of cell cycle regulatory proteins (Wenz et al. 2019). Since it was previously shown that two ferroptosis regulators, TfR1 and ACSL4, are downregulated by E-/N-cadherin-based cell–cell adhesion (Wu et al. 2019), we investigated protein expression of these two proteins in confluent cultures. NIH3T3 cells were seeded to confluence according to our protocol and harvested after 24 and 48 h, respectively. As demonstrated in Fig. 1b, protein expression of TfR1 was stable for at least 24 h and only downregulated 48 h after seeding, while expression of ACSL4 was not decreased at any time point. Similar results were obtained in the human keratinocyte cell line HaCaT and in the colon carcinoma cell line Caco-2 (Supplementary Fig. S1). However, the transcriptional co-regulators YAP/TAZ were excluded from the nucleus demonstrating establishment and functioning of cell–cell contacts (Supplementary Fig. S2). Hence, neither TfR1 nor ACSL4 are downregulated in response to cell–cell contacts at the time point of *t*-BuOOH-exposure, and additional mechanisms must account for cell–cell contact-mediated protection.

### Calcium is required for t-BuOOH-mediated ferroptosis in subconfluent cultures

**Fig. 2:**
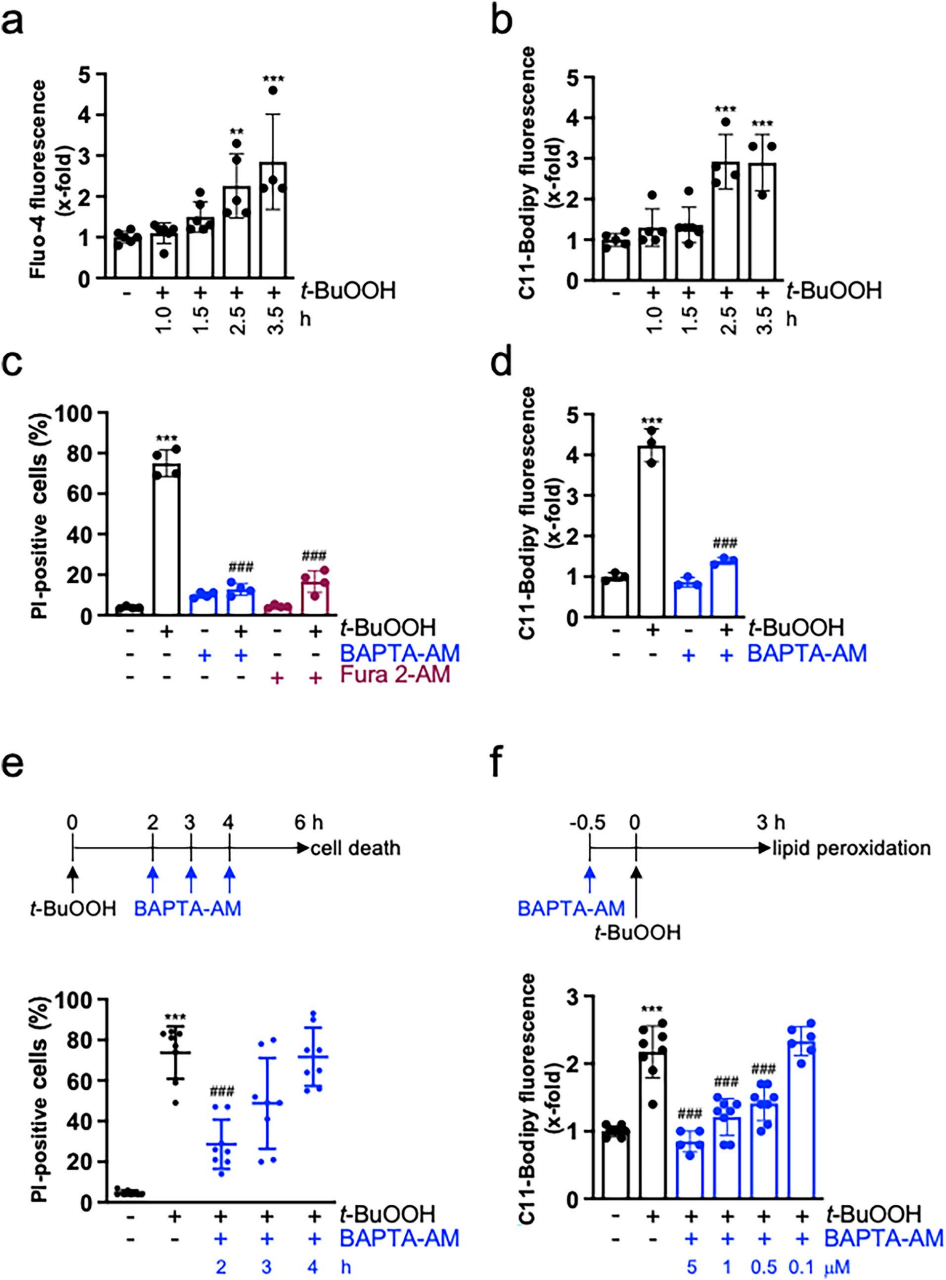
Ca^2+^ is a central regulator of *t*-BuOOH-triggered lipid peroxidation and ferroptosis. NIH3T3 cells were sparsely seeded, cultured for 24 h and treated with *t*-BuOOH (50 µM). **a** Cells were treated with *t*-BuOOH for the indicated time periods. Intracellular Ca^2+^ levels were detected by Fluo-4-fluorescence and flow cytometry. Scatter plot with bars shows means ± SD of x-fold induction of fluorescence relative to untreated controls, *n* = 4–6. **b** Cells were treated with *t*-BuOOH for the indicated time periods. Lipid peroxidation was measured using the lipid peroxidation sensor Bodipy 581/591 C11 and flow cytometry. Scatter plot with bars represents means ± SD of x-fold induction of fluorescence relative to untreated controls, *n* = 3–6. **c** Cells were treated with *t*-BuOOH for 6 h in the absence or presence of BAPTA-AM (5 µM) or Fura 2-AM (10 µM). Cell death was measured as described in Fig. 1a. Scatter plot with bars represent means ± SD of PI-positive cells in %, *n* = 4. **d** Cells were treated with *t*-BuOOH for 3 h in the absence or presence of BAPTA-AM (5 µM). Lipid peroxidation was measured as described in **b**. Scatter plot with bars represent means ± SD of x-fold induction of fluorescence relative to untreated controls, *n* = 3. **e** Cells were treated with *t*-BuOOH for 6 h. BAPTA-AM (5 µM) was added 2, 3, or 4 h after *t*-BuOOH-exposure. Cell death was measured as described in **c**. Scatter plot presents means ± SD of PI-positive cells in %, *n* = 8. **f** BAPTA-AM was added at the indicated concentrations 0.5 h before *t*-BuOOH-exposure, and lipid peroxidation was determined as described in **b**. Scatter blot with bars represents means ± SD of x-fold induction of fluorescence relative to untreated controls, *n* = 5–8. **p < 0.01, ***p < 0.001, significant increase, ###*p* < 0.001, significant inhibition

Since cell–cell contacts prevent not only *t*-BuOOH-triggered ferroptosis, but also loss of MMP and formation of DNA DSBs, we concluded that cell–cell contacts regulate a common mechanism and, vice versa, that one key mechanism is involved in *t*-BuOOH-mediated cellular damage. In view of the known function of Ca^2+^ in cell death and toxicity, we hypothesized that Ca^2+^ might be the central regulator. Indeed, *t*-BuOOH treatment led to a time-dependent increase in intracellular Ca^2+^ levels  ([Ca^2+^]_i_) (Fig. 2a). Interestingly, accumulation of lipid peroxidation was detected at similar time points (Fig. 2b). To show a functional role of Ca^2+^ in ferroptosis, we preincubated the cultures with the cell permeable Ca^2+^-chelator BAPTA-AM. BAPTA-AM extensively blocked cell death supporting our hypothesis that Ca^2+^ is essential for *t*-BuOOH-mediated ferroptosis (Fig. 2c). In line with this conclusion, exposure to BAPTA-AM inhibited *t*-BuOOH-triggered lipid peroxidation to a similar extent (Fig. 2d). To rule out that the inhibitory effect of BAPTA-AM is due to unspecific binding to iron (Britigan et al. 1998), we (i) made use of a second calcium chelator, i.e. Fura 2-AM and (ii) analyzed the effect of BAPTA-AM and the iron chelator deferoxamine on erastin-induced ferroptosis which is heavily dependent on iron (Dixon et al. 2012). The facts that Fura 2-AM also inhibited *t*-BuOOH-triggered ferroptosis (Fig. 2c) and, vice versa, erastin-stimulated ferroptosis was not antagonized by BAPTA-AM, but by deferoxamine [Supplementary Fig. S3, (Dixon et al. 2012)] strongly argue for a specific effect of BAPTA-AM on Ca^2+^.

To show that the increase in [Ca^2+^]_i_ is crucial for *t*-BuOOH-triggered ferroptosis and lipid peroxidation, we added BAPTA-AM at different time points after *t*-BuOOH-exposure (Fig. 2e). Addition of BAPTA-AM 2 h after *t*-BuOOH-treatment still significantly blocked ferroptosis. Addition after 3 h resulted in variable outcomes indicating a point of no return at around 3 h. When BAPTA-AM was added after 4 h, ferroptosis could not be blocked (Fig. 2e). In line, lipid peroxidation was concentration-dependently blocked, when BAPTA-AM was pre-incubated at increasing concentrations 0.5 h before *t*-BuOOH-exposure (Fig. 2f). We conclude that the increase in [Ca^2+^]_i_ is a key factor in *t*-BuOOH-mediated lipid peroxidation and subsequent ferroptosis.

### Calcium is probably released from intracellular stores

**Fig. 3:**
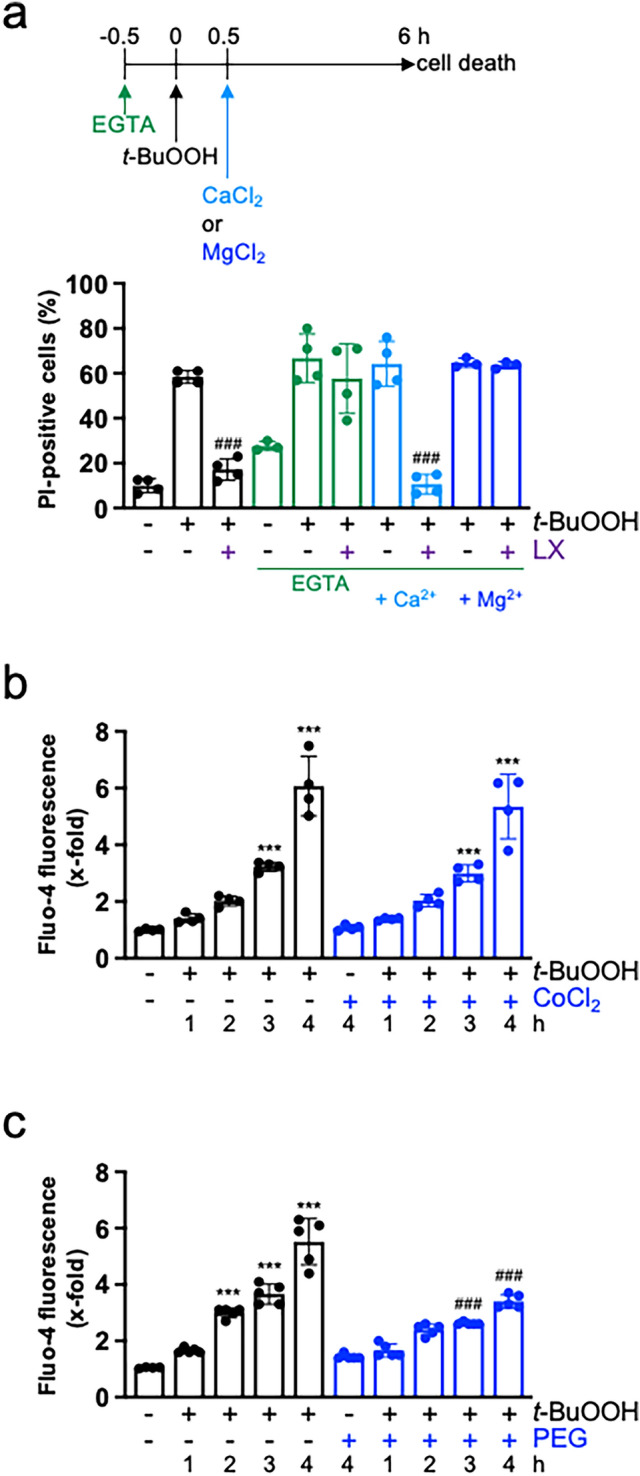
Ca^2+^ is required for *t*-BuOOH-triggered ferroptosis. NIH3T3 cells were sparsely seeded, cultured for 24 h and treated with *t*-BuOOH (50 µM). **a** 30 min prior to *t*-BuOOH-addition, medium was changed to Ca^2+^-free media/10% FBS supplemented with EGTA (0.4 mM). 15 min prior to *t*-BuOOH-exposure, liproxstatin-1 (LX, 1 µM) was added. 30 min after *t*-BuOOH-exposure, Ca^2+^ or Mg^2+^ (each 2 mM) were added and cell death was determined after another 5.5 h as described in Fig. 1a. Scatter plot with bars represents means ± SD of PI-positive cells in %, *n* = 3–4. **b**, **c** Cells were treated with *t*-BuOOH in the absence or presence of CoCl_2_ (20 µM) (**b**) or PEG 8000 (10 mM) (**c**) and harvested at the indicated time points. Intracellular Ca^2+^ levels were detected as described in Fig. 2a. Scatter plot with bars represents means ± SD of x-fold induction of fluorescence relative to untreated controls, *n* = 4–5. ***p < 0.001, significant increase, ###*p* < 0.001, significant inhibition

To support our finding that Ca^2+^ is required for *t*-BuOOH-mediated ferroptosis, we next investigated *t*-BuOOH-mediated cell death in nominally Ca^2+^-free media by addition of EGTA. Interestingly, cell death could still be induced in response to *t*-BuOOH, but could not be blocked by liproxstatin-1 indicating that it was not ferroptosis. Re-addition of Ca^2+^, but not Mg^2+^, 30 min after *t*-BuOOH-exposure re-established ferroptosis (Fig. 3a). Addition of EGTA reduces the availability of extracellular Ca^2+^, but will consequently lead to a decrease in [Ca^2+^]_i_ due to a rapid Ca^2+^ efflux (Borle et al. 1990) (and own unpublished observation). Hence, it was not possible to conclude whether *t*-BuOOH induces Ca^2+^ release from intracellular stores and/or Ca^2+^ influx through Ca^2+^ channels in the plasma membrane. Nevertheless, a twofold increase in [Ca^2+^]_i_ could still be observed after exposure to *t*-BuOOH in Ca^2+^-free media (Supplementary Fig. S4) pointing to a release from intracellular stores. We then made use of the unspecific Ca^2+^ channel blocker CoCl_2_ which inhibits Ca^2+^ influx through channels from the extracellular site (Maher et al. 2018; Tan et al. 1998). *t*-BuOOH-triggered accumulation of [Ca^2+^]_i_ was not inhibited by CoCl_2_ and neither was *t*-BuOOH-triggered ferroptosis nor lipid peroxidation (Fig. 3b, Supplementary Fig. S5a, b). However, it is known that erastin-treatment leads to pore formation in the plasma membrane, Ca^2+^ and water influx from the extracellular sites, finally resulting in cell swelling and lysis. This can be blocked by the osmo-protectant polyethylene glycol (PEG) 8000 (Pedrera et al. 2020; Riegman et al. 2020). To demonstrate that the early increase in [Ca^2+^]_i_ was not the result of *t*-BuOOH-mediated lipid peroxidation and subsequent pore formation, we studied [Ca^2+^]_i_ in the presence of PEG 8000. We detected partial inhibition of the increase in [Ca^2+^]_i_ not earlier than 3 h (Fig. 3c). In line, lipid peroxidation was slightly, but statistically significant reduced 3 h after t-BuOOH-exposure, and ferroptosis was strongly inhibited (Supplementary Fig. S5c,d). We conclude that, in a first step, Ca^2+^ is released from intracellular stores thereby stimulating lipid peroxidation which, in a second step, leads to pore formation in the plasma membrane and further Ca^2+^ influx from the extracellular site.

### Calcium is required for t-BuOOH-induced dissipation of the MMP and DNA DSBs

**Fig. 4:**
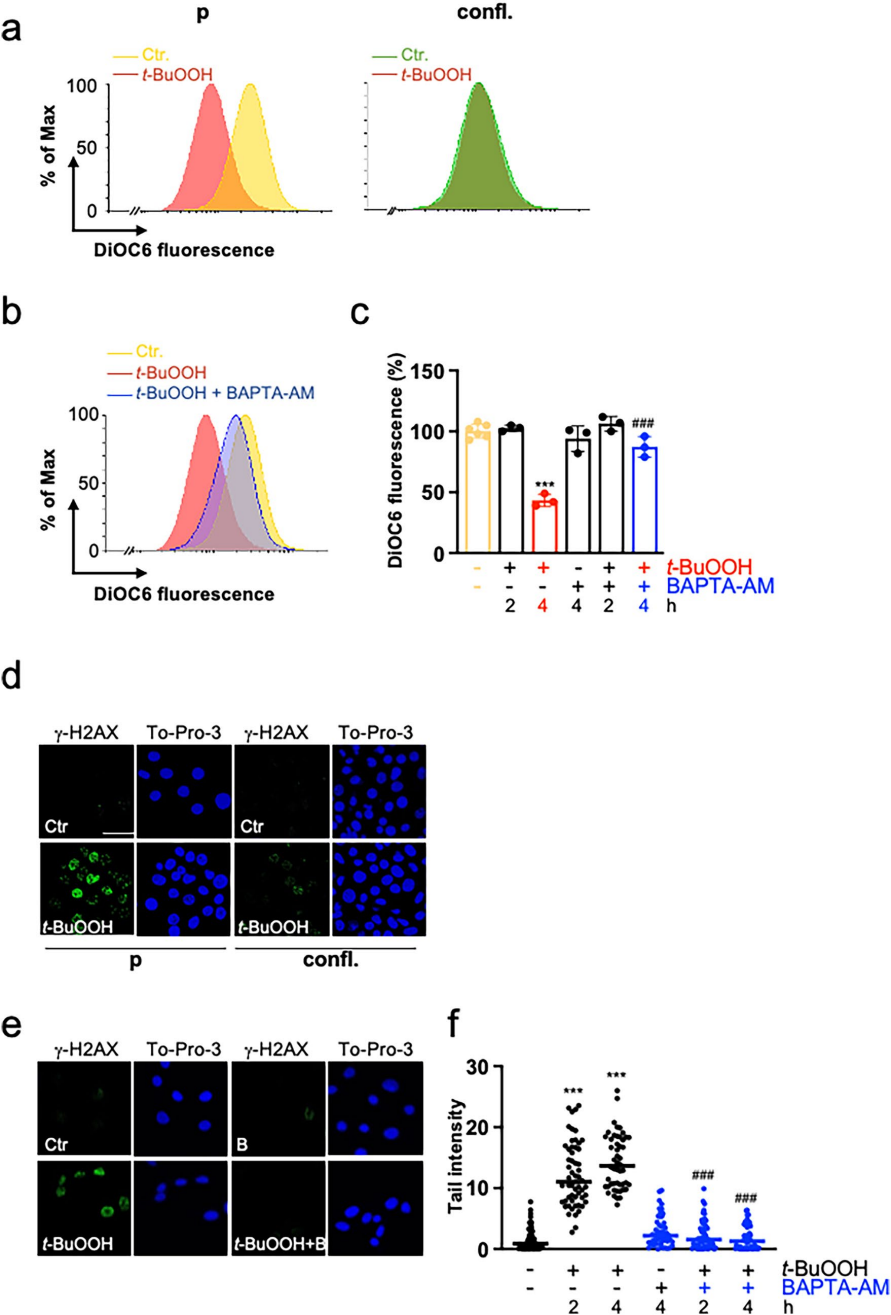
*t*-BuOOH-mediated loss of MMP and DNA DSBs are prevented by cell–cell contacts and require Ca^2+^. **a**, **d** NIH3T3 cells were either sparsely seeded (proliferating = p) or seeded to confluence (confluent = confl.) and cultured for 24 h. *t*-BuOOH (50 µM) was added for 4 h. **b**, **c**, **e**, **f** NIH3T3 cells were sparsely seeded, cultured for 24 h and treated with *t*-BuOOH (50 µM) for 4 h **b**, **e** or 2 and 4 h **c**, **f** in the absence or presence of BAPTA-AM (5 µM). **a**, **b**, **c** Cells were stained with DiOC6 to analyze vitality of mitochondria. Data are shown in a representative histogram out of 3–4 independent experiments **a**, **b** or as a scatter plot with bars showing means ± SD of DiOC6-fluorescence in % of control (= 100%) **c**, *n* = 3–6. **d**, **e**, **f** DNA DSBs were detected by γ-H2AX-immunofluorescence and nuclei counterstained by To-Pro-3 **d**, **e**, or by the neutral Comet assay (**f**). Data are shown in representative pictures **d**, **e** or in tail intensity as scatter plot with median and individual values (analysis of 50 cells) of a representative experiment out 3 independent experiments. B = BAPTA-AM, scale bar = 50 µm, ***p < 0.001, significant effect, ###*p* < 0.001, significant inhibition

We had previously shown that *t*-BuOOH induces a loss of the MMP and DNA DSBs, which is independent of lipid peroxidation and ferroptosis (Wenz et al. 2018). Moreover, cell–cell contacts protect against MMP dissipation and DNA DSBs (Fig. 4a, d) (Wenz et al. 2019). Ca^2+^ overload is well-known to disturb the MMP (Gorlach et al. 2015; Lemasters et al. 2009). In accordance with the data from literature, preincubation with BAPTA-AM prevented *t*-BuOOH-evoked dissipation of the MMP which was analyzed by DiOC6 staining and subsequent flow cytometry (Fig. 4b, c).

Although the mechanism is not clear, it has been shown that Ca^2+^ is required for *t*-BuOOH-triggered DNA single-strand breaks (Guidarelli et al. 1997a, 1997b) (and own unpublished observation). We hypothesized that Ca^2+^ is also essential for *t*-BuOOH-mediated generation of DNA DSBs. Figure 4 demonstrates that BAPTA-AM extensively inhibits formation of DNA DSBs in response to *t*-BuOOH, as assessed by γ-H2AX-staining and the neutral Comet assay (Fig. 4e, f) demonstrating that Ca^2+^ is required for *t*-BuOOH-triggered DNA DSB formation.

Interestingly, cell–cell contacts do not prevent *t*-BuOOH-mediated replication block (Supplementary Fig. S6a) (Wenz et al. 2019). Here we show that the replication block cannot be inhibited by BAPTA-AM indicating that Ca^2+^ is not required (Supplementary Fig. S6b).

### Cell–cell contacts block t-BuOOH-mediated cytosolic increase in calcium

**Fig. 5:**
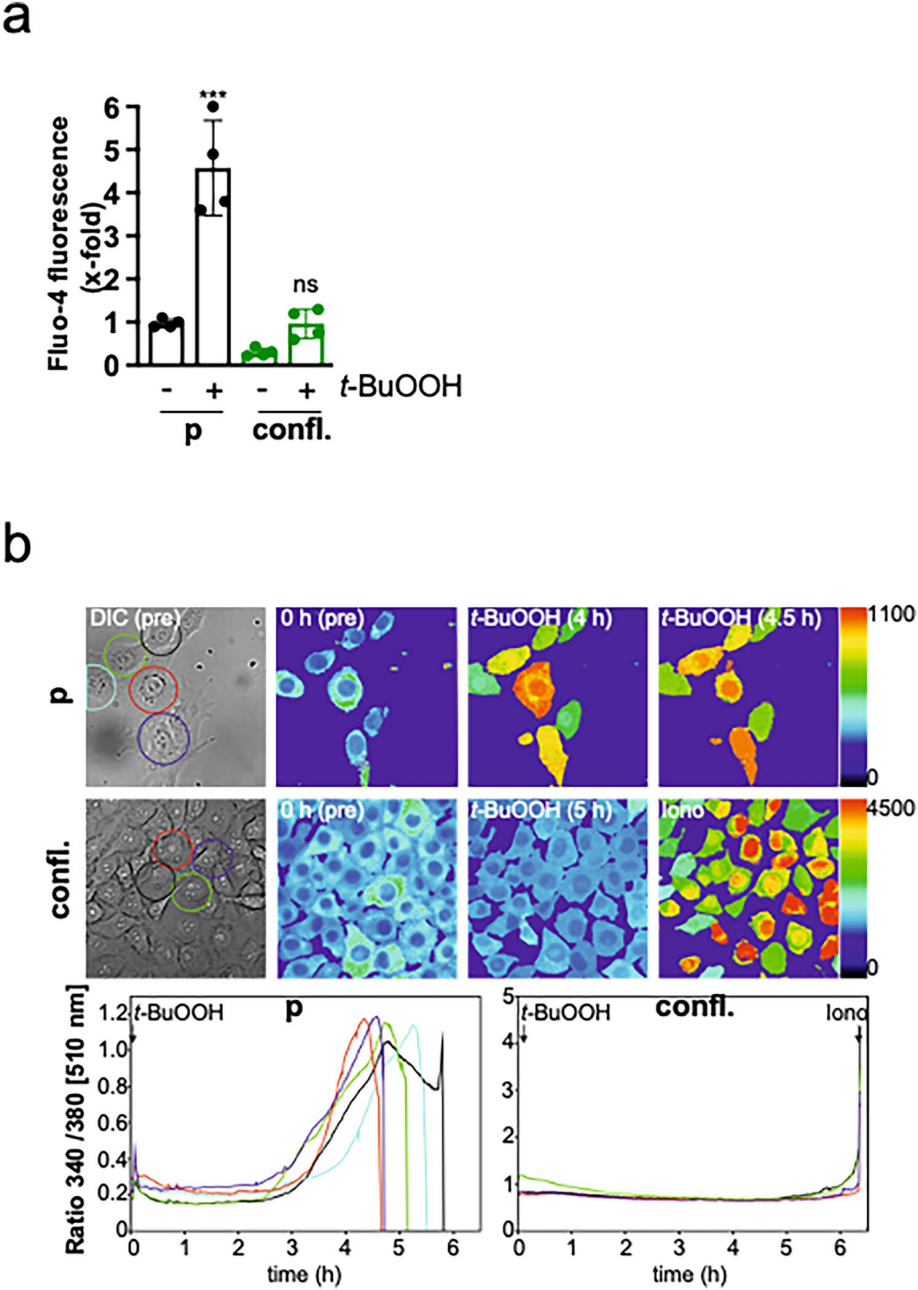
Cell–cell contacts prevent *t*-BuOOH-mediated increase in intracellular Ca^2+^. NIH3T3 cells were either sparsely seeded (proliferating = p) or seeded to confluence (confluent = confl.) and cultured for 24 h. *t*-BuOOH (50 µM) was added for 6 h. **a** Flow cytometry using Fluo-4-AM. Scatter plot with bars represents means ± SD of x-fold induction of fluorescence compared to untreated, proliferating cells, *n* = 4. **b** Live cell imaging using Fura 2-AM. Data are shown as a representative experiment. Vehicle control is presented in Supplementary Fig. S7. Iono = ionomycin (10 µM), DIC = differential interference contrast. *** *p* < 0.001, significant increase, the increase in confluent cultures was not statistically significant (n.s.)

Since we had unraveled a pivotal role of Ca^2+^ in *t*-BuOOH-mediated lipid peroxidation, ferroptosis, MMP dissipation and DNA DSBs, whose effects were prevented by cell–cell contacts, we hypothesized that intracellular Ca^2+^ release might be regulated by cell–cell contacts. Indeed, the *t*-BuOOH-mediated increase in [Ca^2+^]_i_ was strongly inhibited in confluent cultures (Fig. 5a). To confirm our data, live cell imaging using Fura 2-AM was performed. In accordance with our data shown in Fig. 2, [Ca^2+^]_i_ raised in proliferating cultures in response to *t*-BuOOH (Fig. 5b). Here, the increase was detected somewhat later, which can be explained by differences in the experimental settings (cells were kept at room temperature in Tyrode’s solution). The sudden decrease in fluorescence at around 5 h is due loss of the dye as a result of membrane damage. In contrast, no accumulation of [Ca^2+^]_i_ could be detected in confluent cultures. The Ca^2+^ ionophore ionomycin was applied as a positive control for an increase in Ca^2+^.

### Calcium as a central regulator of t-BuOOH-mediated toxicity and regulation by cell–cell contacts in Caco-2 cells

**Fig. 6:**
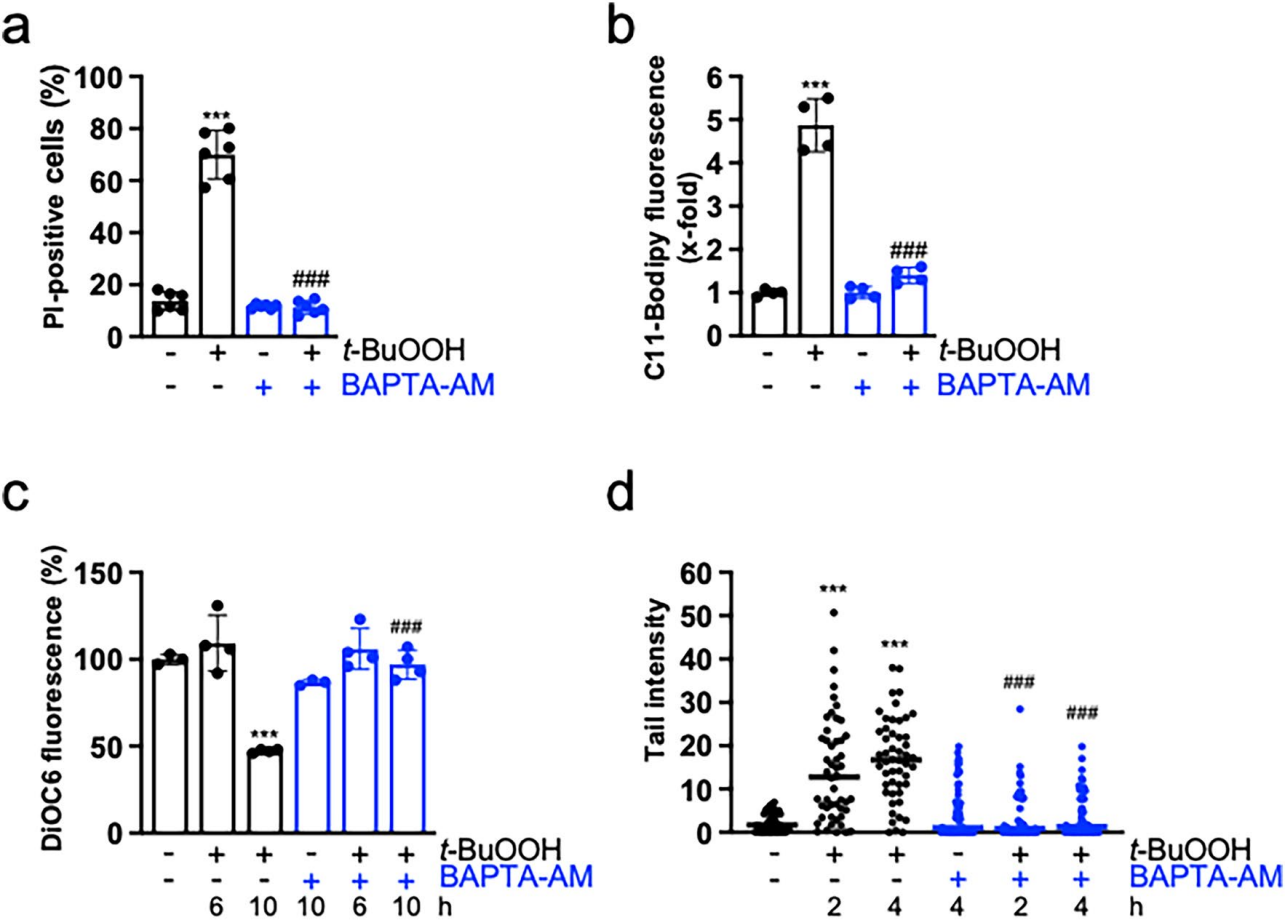
*t*-BuOOH-triggered ferroptosis, lipid peroxidation, loss of MMP and DNA DSBs require Ca^2+^ in Caco-2 cells. Caco-2 cells were sparsely seeded, cultured for 24 h and treated with *t*-BuOOH (100 µM) in the absence or presence of BAPTA-AM (10 µM). **a** Cells were treated for 8 h and cell death analyzed as described in Fig. 1a. Scatter plot with bars represents means ± SD of PI-positive cells in %, *n* = 6. **b** Cells were treated for 3 h and lipid peroxidation was determined as described in Fig. 2b. Scatter plot with bars represents means ± SD of x-fold induction of fluorescence relative to untreated controls, *n* = 4. **c** Cells were treated for the indicated time periods and vitality of the mitochondria was analyzed as described in Fig. 4. Scatter plot with bars shows means ± SD of DiOC6-fluorescence in % of control (= 100%), *n* = 4. **d** Cells were treated for the indicated time periods and DNA DSBs analyzed by the neutral Comet assay. The scatter plot shows median and individual values of tail intensity with 50 cells analyzed per sample of a representative experiment out of 3 independent experiments. ***p < 0.001, significant increase, ###*p* < 0.001, significant inhibition

**Fig. 7:**
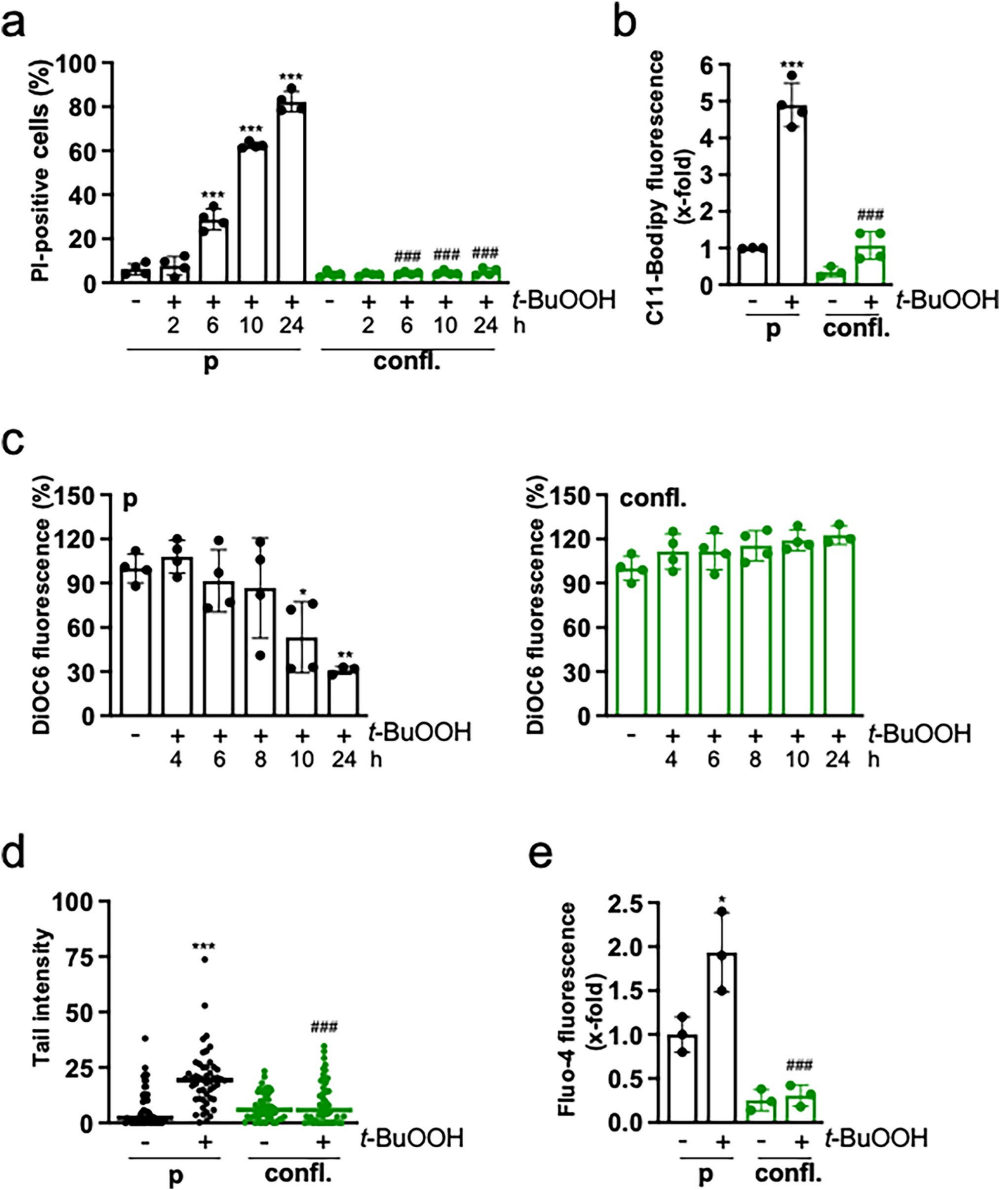
Cell–cell contacts protect against *t*-BuOOH-triggered ferroptosis, lipid peroxidation, loss of MMP and DNA DSBs in Caco-2 cells. Caco-2 cells were either sparsely seeded (proliferating = p) or seeded to confluence (confluent = confl.) and cultured for 24 h. **a** Cells were treated with *t*-BuOOH (100 µM) for the indicated time periods and cell death analyzed as described in Fig. 1a. Scatter plot with bars represents means ± SD of PI-positive cells in %, *n* = 4. **b** Cells were treated with *t*-BuOOH (100 µM) for 3 h and lipid peroxidation was determined as described in Fig. 2b. Scatter plot with bars represents means ± SD of x-fold induction of fluorescence relative to untreated controls, *n* = 3–4. **c** Cells were treated with *t*-BuOOH (100 µM) for the indicated time periods and vitality of mitochondria analyzed as described in Fig. 4a. Scatter plot with bars represents means ± SD of DiOC6-fluorescence in % of control (= 100%), *n* = 4. **d** Cells were treated with *t*-BuOOH (100 µM) for 4 h and DNA DSBs analyzed by neutral Comet assay. The scatter plot shows median and individual values of tail intensity with 50 cells analyzed per sample of a representative experiment out of 3 independent experiments. **e** Caco-2 cells were either sparsely seeded (proliferating = p) or seeded to confluence (confluent = confl.) and cultured for 24 h. *t*-BuOOH (100 µM) was added for 3 h and the increase in intracellular Ca^2+^ was analyzed as described in Fig. 2a. Scatter plot with bars shows means ± SD of fluorescence relative to untreated proliferating controls, *n* = 3. *p < 0.05, **p < 0.01, ***p < 0.001, significant increase, ###*p* < 0.001, significant inhibition

Our data strongly indicated a role of cell–cell contacts in ROS-defense by regulating [Ca^2+^]_i_ at least in untransformed fibroblasts. We finally wanted to know whether this is also true for cancer cells and focused our interest on the colon carcinoma cell line Caco-2. As seen in fibroblasts, *t*-BuOOH induces ferroptosis in Caco-2 cells (Supplementary Fig. S8a), and ferroptosis, lipid peroxidation, loss of MMP and DNA DSB formation require Ca^2+^ (Fig. 6a–d). Moreover, confluent Caco-2 cells are protected against *t*-BuOOH-triggered ferroptosis (Fig. 7a, Supplementary Fig. S8b). Cell–cell contacts also prevent *t*-BuOOH-mediated lipid peroxidation (Fig. 7b), dissipation of the MMP (Fig. 7c) and DNA DSBs (Fig. 7d). In line with our observations in fibroblasts, exposure to *t*-BuOOH results in an increase in [Ca^2+^]_i_ in subconfluent cultures which is prevented in confluent Caco-2 cells (Fig. 7e).

**Fig. 8:**
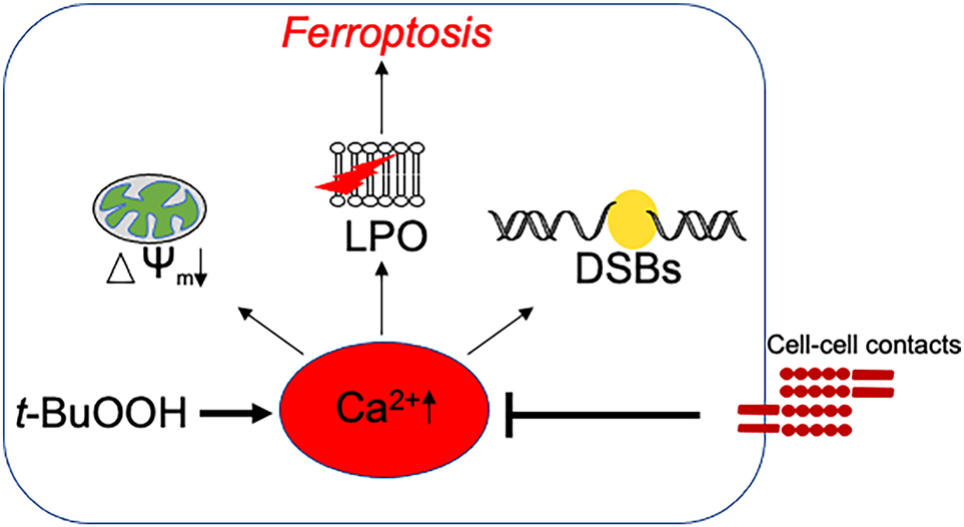
Proposed model of cell–cell contact-mediated protection against ROS-induced cellular damage. *t*-BuOOH leads to an increase in intracellular Ca^2+^ which is required for lipid peroxidation and subsequent ferroptosis, loss of the mitochondrial membrane potential and DNA double strand breaks. Cell–cell contacts prevent the accumulation of intracellular Ca^2+^. Since Ca^2+^ is a key mediator of the oxidative stress response and of various cell death pathways, our observations explain the broad protective function of cell–cell contacts against a variety of exogenous toxicants

We provide the model (Fig. 8) that cell–cell contacts inhibit Ca^2+^ release from intracellular organelles, thereby preventing *t*-BuOOH-mediated lipid peroxidation, ferroptosis, dissipation of the MMP and DNA DSBs.

## Discussion

Here, we describe a central role of Ca^2+^ in *t*-BuOOH-mediated toxicity, i.e., lipid peroxidation with subsequent ferroptosis, loss of MMP and formation of DNA DSBs. We further present Ca^2+^ as a key mediator of protection against these effects by cell–cell contacts. This conclusion is based on the following observations: (i) intracellular Ca^2+^ levels are increased in response to *t*-BuOOH, (ii) *t*-BuOOH-mediated cellular damage, i.e. lipid peroxidation, MMP dissipation, and DNA DSBs, is blocked by the cell-permeable Ca^2+^ chelator BAPTA-AM, and (iii) cell–cell contacts inhibit the increase in intracellular Ca^2+^, and, at the same time, induction of damage. Vice versa, *t*-BuOOH-triggered replication block is not blocked by BAPTA-AM (present work) indicating independence of Ca^2+^, and it is not affected by cell–cell contacts (present work) (Wenz et al. 2019).

### Mechanism of t-BuOOH-triggered increase in intracellular Ca^2+^

It is known that oxidative stress, including *t*-BuOOH, may lead to an increase in [Ca^2+^]_i_ (Gorlach et al. 2015; Sakaida et al. 1991). Hence, our observation of an accumulation of [Ca^2+^]_i_ in response to *t*-BuOOH was not unexpected. However, the role of Ca^2+^ in *t*-BuOOH-mediated toxicity as well as the mechanism(s) of *t*-BuOOH-triggered increase in [Ca^2+^]_i_ remain to be elucidated (Sakaida et al. 1991). The elevation of [Ca^2+^]_i_ could be due to (i) influx from the extracellular space through plasma membrane Ca^2+^ channels (Hempel and Trebak 2017), (ii) a release from intracellular Ca^2+^ stores and/or inhibition of efflux as a result of oxidation of channels and/or pumps (Gorlach et al. 2015; Hempel and Trebak 2017), (iii) opening of the mitochondrial permeability transition pore (mPTP, see below), and/or (iv) secondary to plasma membrane damage. Since the unspecific Ca^2+^ channel blocker CoCl_2_ (Maher et al. 2018; Tan et al. 1998) did not prevent the increase in [Ca^2+^]_i_, neither lipid peroxidation nor ferroptosis, an uptake of Ca^2+^ from the extracellular space through plasma membrane Ca^2+^-channels is unlikely. Significant accumulation of [Ca^2+^]_i_ was already measured 2 h after *t*-BuOOH-exposure which further increased in a time-dependent manner. We know that the MMP is disturbed later, i.e. at about 3 h (Wenz et al. 2019), and that loss of MMP is blocked by BAPTA-AM (present work). We conclude that Ca^2+^ is first released from intracellular stores and/or that efflux is inhibited, which then leads to Ca^2+^ overload in the mitochondria with subsequent release through opening of the mPTP (Gorlach et al. 2015). In line with our assumption, it has been shown that *t*-BuOOH modifies the ryanodine receptor (Martinez-Burgos et al. 2006), but oxidation of the IP3-receptor has also been demonstrated (Bird et al. 1993; Poirier et al. 2001). Both the ryanodine receptor and the IP3-receptor are activated by oxidation, hence prompting Ca^2+^ release. Preliminary experiments suggest that Ca^2+^ is indeed partially released from the ER by the IP3-receptor. Although still not demonstrated for *t*-BuOOH, it is known that the endoplasmic Ca^2+^-ATPase SERCA is also redox-sensitive (Redondo et al. 2004). Furthermore, ROS may inhibit export of Ca^2+^, since the Na^+^/Ca^2+^ exchanger can be reversed at high ROS concentrations, and plasmalemmal Ca^2+ //^ATPase can be inhibited (Nicotera et al. 1985)(for review see Ermak and Davies 2002; Hempel and Trebak 2017). Finally, it is known that erastin-induced ferroptosis is executed by Ca^2+^ influx from the extracellular site, which is mediated by pores built in response to lipid peroxidation (Pedrera et al. 2020; Riegman et al. 2020). In line with these findings, the osmoprotectant PEG 8000 prevented *t*-BuOOH-triggered ferroptosis and reduced [Ca^2+^]_i_ at later time points (> 3 h after *t*-BuOOH-exposure). We conclude that Ca^2+^ accumulation occurs as a trigger event inducing lipid peroxidation, and as a secondary event (> 3 h) downstream of lipid peroxidation. We assume that the early (< 3 h) increase in [Ca^2+^]_i_ is due to a release from intracellular stores and/or inhibition of export. Around and after the point of no return (about 3 h after *t*-BuOOH-addition), Ca^2+^ is released from the mitochondria due to collapse of the MMP, and, thereafter and even more importantly, Ca^2+^ enters the cell through pores. The precise origin of intracellular Ca^2+^ upstream of lipid peroxidation is currently under investigation.

### Role of Ca^2+^ in t-BuOOH-mediated dissipation of the MMP

When intracellular Ca^2+^ raises, it is sequestered by the mitochondria. Ca^2+^ overload promotes opening of the mitochondrial permeability transition pore (mPTP), a high conductance channel. Prolonged opening of the mPTP causes an increase in permeability of the inner mitochondrial membrane to ions and solutes up to masses of 1500 kD. This finally leads to a collapse of the MMP, disruption of the outer mitochondrial membrane, release of Ca^2+^ and proteins and may, subsequently, cause apoptosis or MPTP-driven necrosis (Lemasters et al. 2009; Rasola and Bernardi 2011). It is important to note that, in our cell system, loss of MMP does not lead to apoptosis or MPTP-driven cell death and is not involved in *t*-BuOOH-triggered ferroptosis (Wenz et al. 2018). In line with this finding, inhibition of the mitochondrial calcium uniporter (MCU) by ruthenium red, which, beyond several other mechanisms, blocks mitochondrial uptake of Ca^2+^, does not inhibit, but rather increases lipid peroxidation and ferroptosis (unpublished observation).

### Role of Ca^2+^ in t-BuOOH-mediated lipid peroxidation

Our data clearly show that Ca^2+^ is required for lipid peroxidation in *t*-BuOOH-mediated ferroptosis. This is in contrast to erastin-treatment (Dixon et al. 2012) and hints at a different mechanism. The role of Ca^2+^ in *t*-BuOOH-mediated lipid peroxidation is not understood yet. To the best of our knowledge, Ca^2+^ is not required for the initiation or propagation of lipid peroxidation itself (Cheng and Li 2007). Hence, it presumably plays a role as a ROS stimulator. As stated above, *t*-BuOOH-mediated ferroptosis is independent of MMP dissipation. Nevertheless, a role of the mitochondria preceding loss of MMP is plausible. An increase in Ca^2+^ stimulates mitochondrial electron transport activity and thereby superoxide production (Castilho et al. 1995; Gorlach et al. 2015; Guidarelli et al. 1997b). (The calcium binding site of enzymes, such as e.g. FAD-glycerol phosphate dehydrogenase, or 2-oxoglutarate dehydrogenases lay outside the inner mitochondrial membrane). This is converted to hydrogen peroxide which can freely diffuse to any cell organelle including cell membranes. Here, hydrogen peroxide can react in the presence of iron to the hydroxyl radical and/or to different iron/oxo species, thereby initiating lipid peroxidation (Cheng and Li 2007; Prescott and Bottle 2017). Our finding that *t*-BuOOH-triggered ferroptosis is blocked by the mitochondria-specific ROS scavenger XJB-5–131 (Krainz et al. 2016; Wenz et al. 2018) is consistent with the idea of mitochondrial ROS-production. In addition, several ROS-generating enzymes, such as lipoxygenases (Czapski et al. 2016), NADPH-oxidases (Brandes et al. 2014), NO synthase (Alderton et al. 2001) and/or xanthine oxidases (Vickneson 2020), are activated by Ca^2+^. Preliminary results speak against a role of lipoxygenases (unpublished observation). Further research is needed to elucidate the role of mitochondria and/or these enzymes in *t*-BuOOH-mediated lipid peroxidation.

### Role of Ca^2+^ in t-BuOOH-mediated induction of DNA DSBs

The precise role of Ca^2+^ in formation of DNA DSBs also remains to be elucidated. Lipid peroxidation end products, such as malondialdehyde or 4-hydroxynonenal only partially account for DNA DSB formation (Wenz et al. 2018). It is further possible that Ca^2+^ activates mitochondrial superoxide and subsequent hydrogen peroxide formation, as described above. Such a mechanism concerning *t*-BuOOH-mediated generation of DNA SSBs (Guidarelli et al. 1997b) has already been proposed. Since hydrogen peroxide can freely diffuse, it may enter the nucleus where it is further metabolized to the hydroxyl radical by the Fenton reaction. This hypothesis is currently being investigated in our group. Additionally, it is possible that Ca^2+^ stimulates endonucleases (Ueda and Shah 1992).

### Role of ACSL4 and TfR1 in protection by cell–cell contacts

Our most important finding is that cell–cell contacts protect against *t*-BuOOH-mediated toxicity by regulation of [Ca^2+^]_i_, but not by ACSL4 or TfR1. The cell adhesion molecule mediating protection is not known so far. In an elegant work, Wu and coworkers had previously demonstrated, that E/N-cadherin activate the Hippo pathway via Merlin, thereby leading to nuclear export of YAP and hence transcriptional downregulation of ACSL4 and TfR1, two important regulators of ferroptosis (Wu et al. 2019). However, expression of YAP/TAZ target genes depends on the context (van Soldt and Cardoso 2020). We could not detect any decrease in ACSL4 in three different confluent cell culture models indicating cell type specificity of cell density-dependent regulation of ACSL4. Such a cell-type specificity is in line with previous observations demonstrating ACSL4 regulation by a multitude of regulators and different transcription factors beyond YAP in a cell- and tissue-dependent manner (Orlando et al. 2013). We also could not detect downregulation of TfR1 protein 24 h after seeding, the time point of *t*-BuOOH-exposure. Only in NIH3T3 cells, a decrease in TfR1 protein levels could be observed at a later time point. The discrepancy of the results seen by us and others can be explained by (i) cell-type specificity, since other transcription factors might also contribute to TfR1 expression (Lu et al. 2021) and / or (ii) slight differences in the density the cells were seeded and hence the time point when signaling via cell–cell adhesion starts. At least, our finding of a later TfR1-downregulation in NIH3T3 cells is in accordance with its described protein half-life of 23 ± 4 h (Rutledge et al. 1991). Whether N/E-Cadherin is involved in regulation of [Ca^2+^]_i_ remains to be elucidated. We also do not know about the signaling cascade and the intracellular target(s) finally leading to the inhibition of the increase in [Ca^2+^]_i_. To note, erastin-induced ferroptosis is also prevented in our cell culture system, but it does not require Ca^2+^ for lipid peroxidation. Hence, additional mechanisms may account for cell–cell contact-mediated protection.

## Conclusions

We have shown that *t*-BuOOH-treatment leads to lipid peroxidation and ferroptosis as well as loss of MMP and formation of DNA DSBs. These toxic outcomes totally depend on *t*-BuOOH-induced increase in intracellular Ca^2+^. Cell–cell contacts not only prevent ferroptosis and *t*-BUOOH-triggered formation of DNA DSBs and dissipation of the MMP, but also protect against hydrogen peroxide, methyl methanesulfonate as well as UV-C-mediated cell death (Wenz et al. 2019). Here, we provide novel insights into the underlying mechanism and propose a novel model in which cell–cell contacts control Ca^2+^ release from intracellular organelle(s) and/or Ca^2+^ efflux.

Since Ca^2+^ is a central player of toxicity in response to oxidative stress and is involved in various cell death pathways, our observation explains the broad protective function of cell–cell contacts against a variety of exogenous toxicants.

### Supplementary Information

Below is the link to the electronic supplementary material.

Supplementary file1 (PDF 4649 KB)

Supplementary file2 (PDF 2151 KB)

## Data Availability

The datasets used and/or analyzed to support the findings of this study are available in this article or in the Supplementary Figures. All the raw data supporting the results of this study are available from the corresponding author upon request.
