# Supplementary material for: Cell–cell contacts prevent t-BuOOH-triggered ferroptosis and cellular damage in vitro by regulation of intracellular calcium
Source: Arch Toxicol. 2024 May 30;98(9):2953–69. doi: 10.1007/s00204-024-03792-5 (PMC11324706; doi:10.1007/s00204-024-03792-5)
Supplement: Supplementary file 1 — Supplementary file1 (PDF 4649 KB) [file 204_2024_3792_MOESM1_ESM.pdf]

Supplementary Material

**Cell-cell contacts prevent *t*-BuOOH-triggered ferroptosis and cellular damage *in vitro* by regulation of intracellular calcium**

Dagmar Faust, Christine Wenz, Stefanie Holm, Gregory Harms, Wolfgang Greffrath, Cornelia Dietrich*

Institute of Toxicology, University Medical Center of the Johannes Gutenberg University, Obere Zahlbacher Straße 67, 55131 Mainz, Germany

*Corresponding author:

Cornelia Dietrich
Institute of Toxicology
University Medical Center of the Johannes Gutenberg-University
Obere Zahlbacherstr. 67
55131 Mainz
Germany
e-mail: cdietric@uni-mainz.de

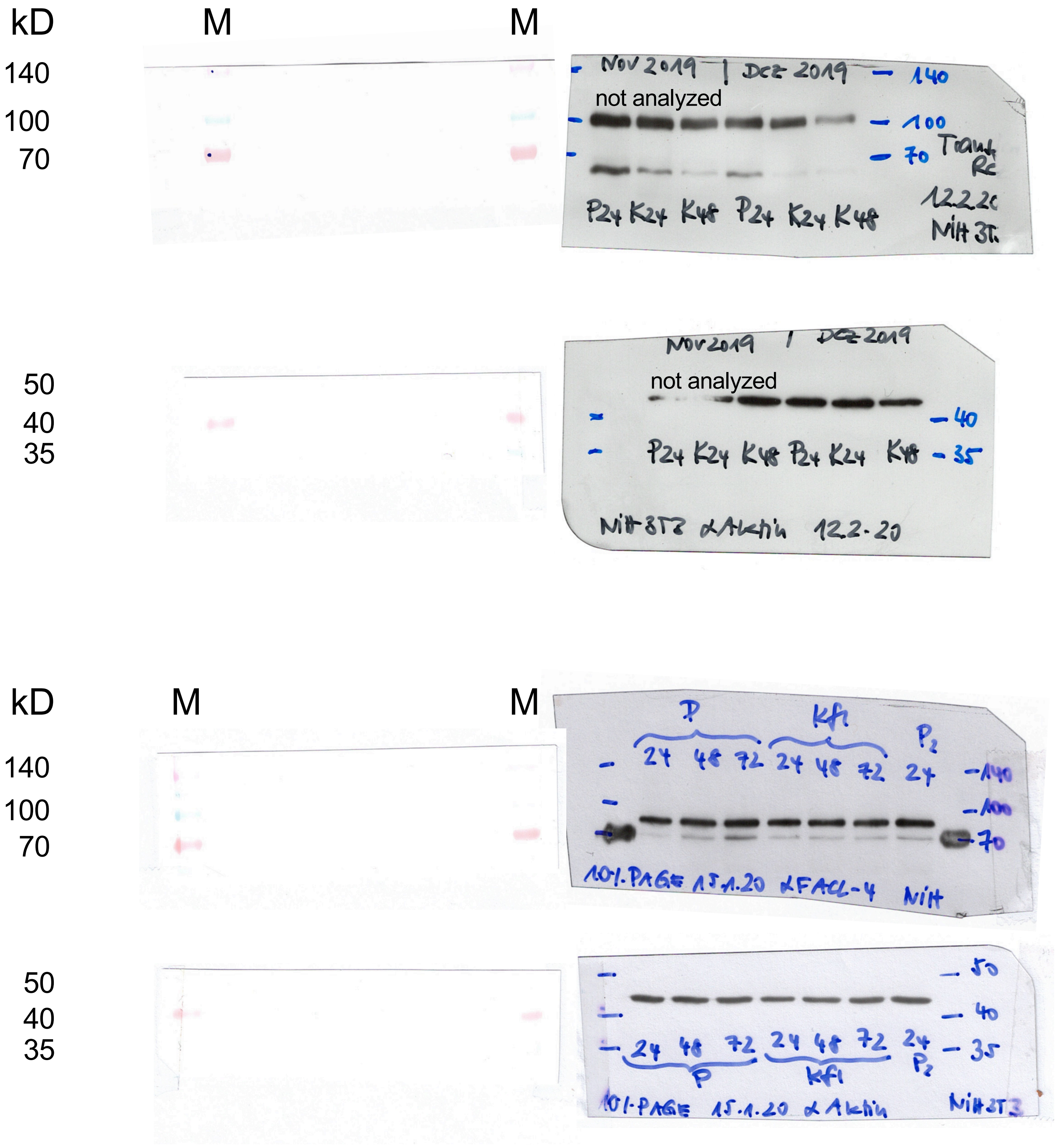


**Original Blots to Fig. 1** Blots were cut in upper and lower part to detect protein of interest and ß-actin (loading control) on the same blot and to avoid stripping.
p = proliferating, k / kfl = confluent, M = marker, FACL-4 = ACSL4
