# Supplementary material for: Cell–cell contacts prevent t-BuOOH-triggered ferroptosis and cellular damage in vitro by regulation of intracellular calcium
Source: Arch Toxicol. 2024 May 30;98(9):2953–69. doi: 10.1007/s00204-024-03792-5 (PMC11324706; doi:10.1007/s00204-024-03792-5)
Supplement: Supplementary file 2 — Supplementary file2 (PDF 2151 KB) [file 204_2024_3792_MOESM2_ESM.pdf]

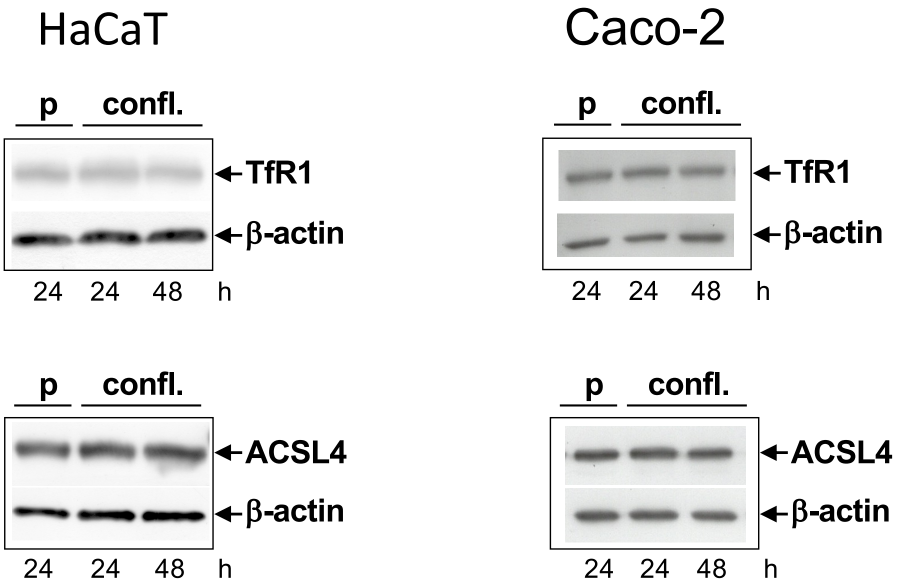

Supplementary Fig. S1 TfR1 and ACSL4 are not downregulated in confluent HaCaT or Caco-2 cells. Cells were either sparsely seeded (proliferating = p) and cultured for 24 h or seeded to confluence (confluent = confl.) and cultured for 24 and 48 h. Total cell extracts were prepared and Western blot performed using an anti-TfR1- or anti-ACSL4-antibody. Blots were stripped and reprobed with anti-β-actin-antibody to control equal loading.

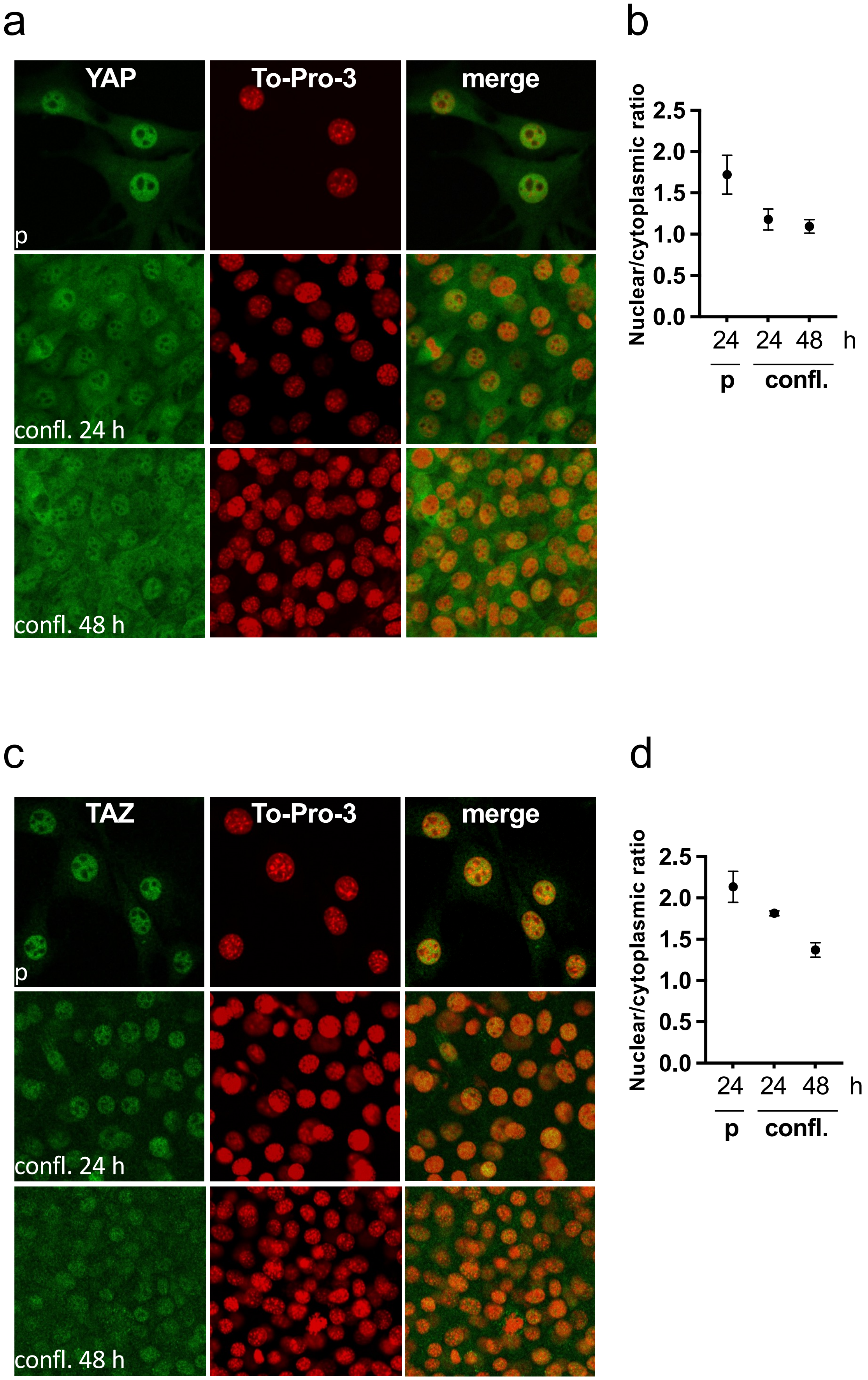
a
YAP
To-Pro-3
merge
p
confl. 24 h
confl. 48 h
b
Nuclear/cytoplasmic ratio
2.5
2.0
1.5
1.0
0.5
0.0
24
24
48
h
p
confl.
c
TAZ
To-Pro-3
merge
p
confl. 24 h
confl. 48 h
d
Nuclear/cytoplasmic ratio
2.5
2.0
1.5
1.0
0.5
0.0
24
24
48
h
p
confl.

Supplementary Fig. S2 YAP/TAZ are excluded from the nucleus in confluent NIH3T3 cells. Cells were either sparsely seeded (proliferating = p) and cultured for 24 h or seeded to confluence (confluent = confl.) and cultured for 24 and 48 h. After fixation with 4 % paraformaledyhde and permeabilization with 0,2 % Triton-X-100 / PBS, cells were blocked with PBS containing 5 % Albumin Fraction V. Cells were then incubated with anti-YAP- (a,b) or anti-TAZ-antibody (c,d). Nuclei were counterstained with To-Pro-3. Representative confocal images were captured by a Leica SP8 laser scanning microscope with a 20X 0.75 objective with 488 nm (Alexa 488) and 638 nm (To-Pro-3) excitation, simultaneously (a,c). Quantitative analysis (b,d) was performed by importing the images in Imaris (version 9.3.3, Bitplane, Zurich, Switzerland) with automated detection of the nucleus from the To-Pro-3 image and from the cell border from the Alexa 488 image. From each cell, the average fluorescence signal of the Alexa 488 fluorescence in the nuclear and cytosol was quantified, and the nucleus to cytosol ratio of the Alexa 488 signal was determined per cell. The average and standard deviation is depicted under each condition in the graphs.

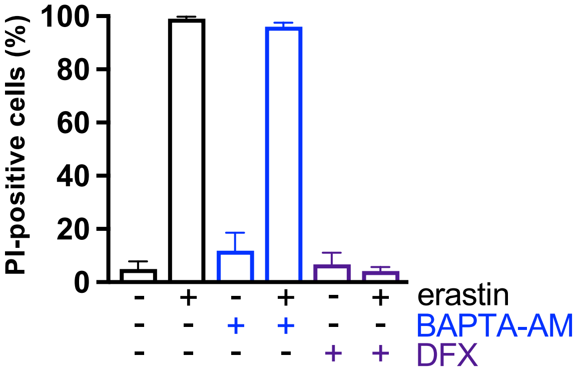

Supplementary Fig. S3 Erastin-triggered ferroptosis is blocked by deferoxamine, but not by BAPTA-AM**.** NIH3T3 cells were sparsely seeded, cultured for 24 h and treated with erastin (10 µM) for 24 h in the absence or presence of BAPTA-AM (5 µM) or deferoxamine (DFX, 100 µM). Cell death was determined by PI-staining and flow cytomery. Bars represent means±SD of PI-positive cells, n= 4.

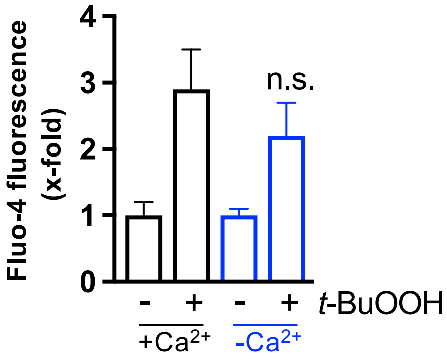

Supplementary Fig. S4 Intracellular calcium increases in calcium-free media in response to *t*-BuOOH. NIH3T3 cells were sparsely seeded and cultured for 24 h. Cells were washed and calcium-free media was added (-Ca^{2+}). Cells were treated with *t*-BuOOH for 3 h and Ca^{2+} was measured using Fluo-4 and flow cytometry. Bars represent means±SD of x-fold induction relative to untreated controls, n=4. The induction in the absence of extra-cellular Ca^{2+} (-Ca^{2+}) was not different to the induction in the presence of extracellular Ca^{2+} (+Ca^{2+}) .

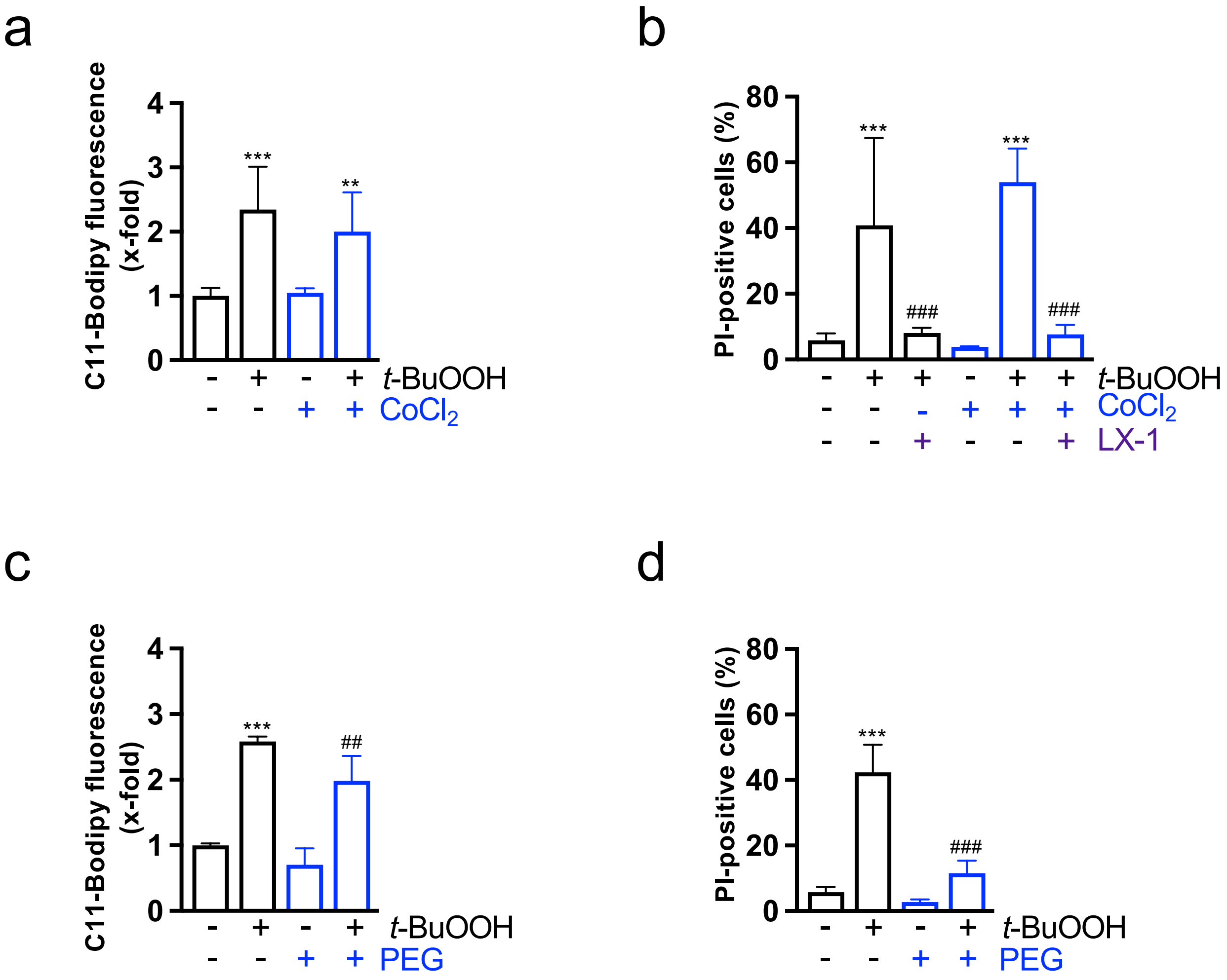

Supplementary Fig. S5 Effect of $CoCl_2$ and PEG 8000 on lipid peroxidation and ferroptosis. NIH3T3 cells were sparsely seeded and cultured for 24 h. (a,c) Cells were treated with *t*-BuOOH for 3 h in the absence or presence of $CoCl_2$ (a) or PEG 8000 (PEG) (c) and lipid peroxidation was measured using Bodipy 581/591 C11 and flow cytometry. Bars represent means±SD of x-fold induction relative to untreated controls, n=4. The induction of lipid peroxidation in the presence of $CoCl_2$ was not different to the induction in the absence of $CoCl_2$. (b,d) Cells were treated with *t*-BuOOH for 6 h in the absence or presence of $CoCl_2$ (b) or PEG8000 (PEG) (d) and cell death was measured by PI staining and flow cytometry. Bars represent means±SD of PI-positive cells, n=4. The induction of ferroptosis in the presence of $CoCl_2$ was not different to the induction in the absence of $CoCl_2$. To confirm ferroptosis, cell death was additionally analyzed in the absence or presence of liproxstatin-1 (LX-1, 1 µM).

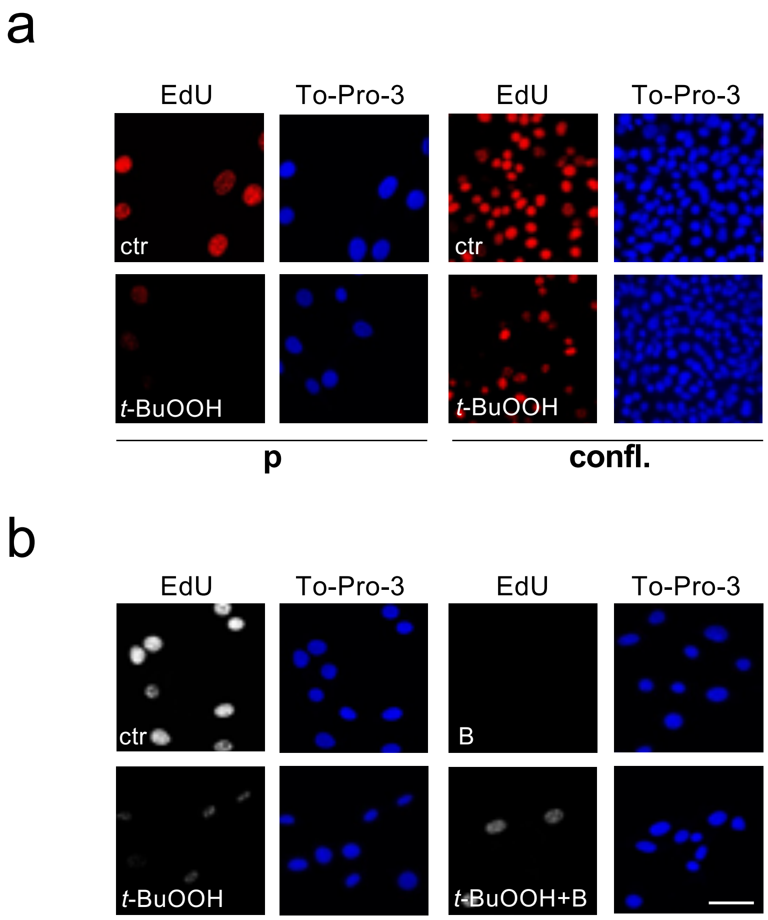

Supplementary Fig. S6 *t*-BuOOH-mediated replication block is not altered by cell-cell contacts and does not require Ca^{2+}. (a) NIH3T3 cells were either sparsely seeded or seeded to confluence and cultured for 24 h. Cells were treated with *t*-BuOOH for 4 h. (b) Cells were sparsely seeded and cultured for 24 h. Cells were treated with *t*-BuOOH for 4 h in the absence or presence of BAPTA-AM. Replication block was detected by the Click-iT™ EdU Imaging Kit. Nuclei were counterstained with To-Pro-3. B=BAPTA-AM, scale bar = 50 µm

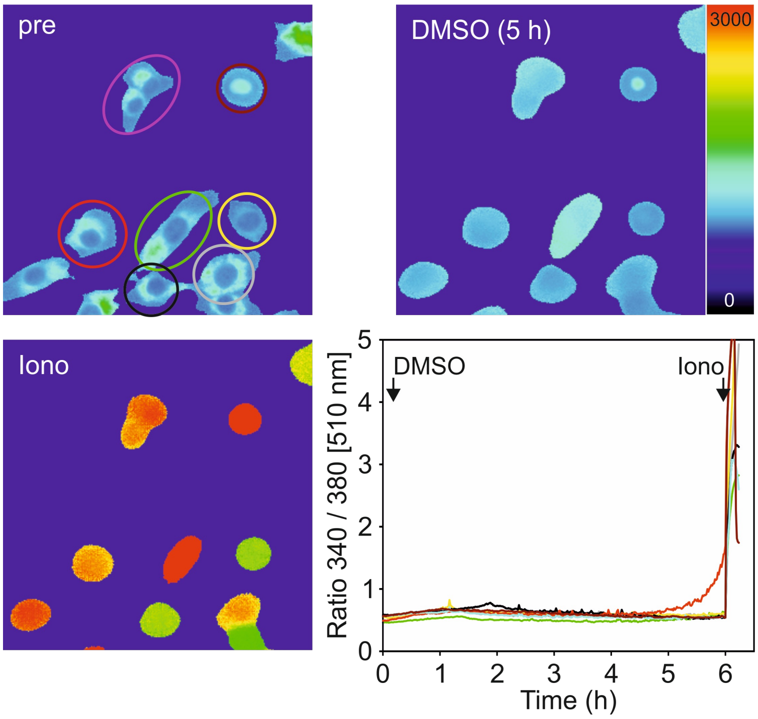

Supplementary Fig. S7 Vehicle control to Fig. 5. NIH3T3 cells were sparsely seeded and treated with DMSO. Live cell imaging was performed using Fura 2-AM. Iono = ionomycin

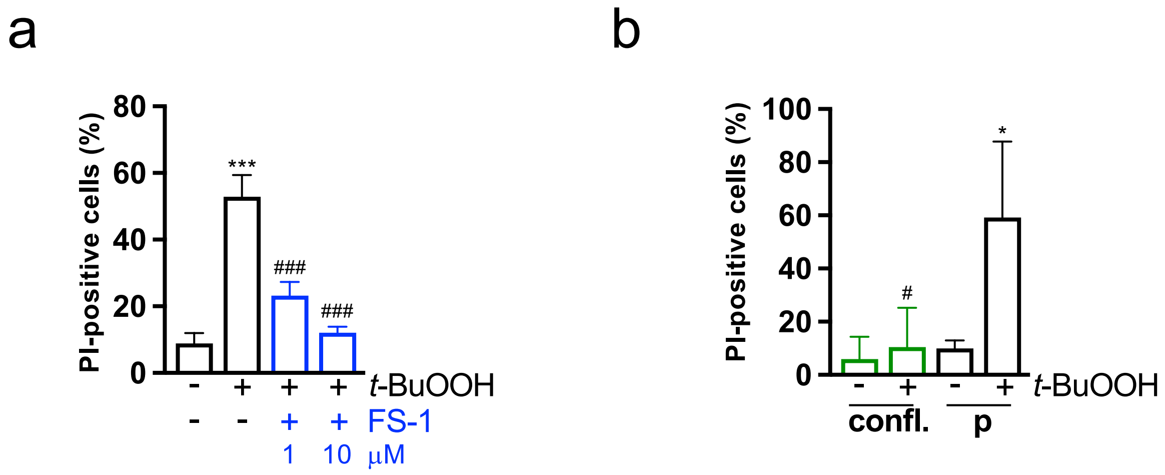

Supplementary Fig. S8 *t*-BuOOH induces ferroptosis in Caco-2 cells which is prevented in confluent cultures. (a) Caco-2 cells were sparsely seeded and cultured for 24 h. Cells were treated with *t*-BuOOH (100 µM) for 6 h in the absence or presence of ferrostatin-1 (FS-1). (b) Cells were either sparsely seeded (proliferating = p) or the same amount of cells was seeded in a small volume as a drop to create confluent cultures (confl.) as previously described (Wenz et al., 2019). (a, b) Cell death was determined by PI-staining and flow cytometry. Bars represent means±SD of x-fold induction relative to untreated controls, n=4
